# Effects of *VeA* Gene on the Growth, Pigment and Citrinin Synthesis of *Monascus ruber* M7

**DOI:** 10.3390/microorganisms14010137

**Published:** 2026-01-08

**Authors:** Linsha Kuang, Peng Ma, Xia He, Liling Wang, Qiuye Xie, Yi He, Huilin Tan

**Affiliations:** 1 Production & Construction Group Key Laboratory of Special Agricultural Products Further Processing in Southern Xinjiang, College of Food Science and Engineering, Tarim University, Alar 843300, China; klsdyx1252191220@163.com (L.K.); mp940112@126.com (P.M.); 13408250568@163.com (X.H.); m17877425543@163.com (Q.X.); 2 National R&D Center for Se-Rich Agricultural Products Processing, Hubei Engineering Research Center for Deep Processing of Green Se-Rich Agricultural Products, School of Modern Industry for Selenium Science and Engineering, Wuhan Polytechnic University, Wuhan 430023, China; yi.he@whpu.edu.cn; 3 Aksu Prefecture Inspection and Testing Center, Aksu 843000, China; 19183467827@163.com

**Keywords:** *Monascus*, *VeA* gene, gene knockout, pigment, citrinin

## Abstract

*Monascus* spp. is widely used in food fermentation and additive production, and some of its strains produce citrinin (CIT), which is nephrotoxic. In this study, we constructed a mutant strain of *Monascus ruber* M7 (*M. ruber* M7) with a *VeA* gene deletion (*ΔVeA*) using the *Agrobacterium*-mediated transformation technique to investigate the *VeA* gene in growth and development, pigmentation, and CIT synthesis in *M. ruber* M7. Compared to the wild-type strain (WT), the *ΔVeA* strain grew faster, the mycelium was dense, the length was longer, the colony color was lighter, the cleistothecium and conidia were reduced, the ability to produce *Monascus* pigments (MPs) decreased, and CIT was not produced. Transcriptomic results showed that differentially expressed genes (DEGs) were mainly enriched in amino sugar metabolism, the MAPK signaling pathway and carbohydrate metabolism. These results suggest that the *VeA* gene negatively regulates the growth and development of *M. ruber* M7 and positively regulates its sexual/asexual reproduction, MPs, and CIT production. The results of this study can provide a reference for industrial applications of *Monascus* with low or even no CIT production.

## 1. Introduction

*Monascus* spp. is widely found in barley, corn, wheat, nuts, silage, kimchi, and other crops and substrates [1,2]. *Monascus* spp. can produce a large number of beneficial primary and secondary metabolites, such as MPs, Monacolin K, gamma-aminobutyric acid (GABA), saccharase, esterase, protease, and other enzyme-active substances [3,4]. It plays an important role in the fields of food additives production, medicine [5,6,7,8].

In 1995, Blanc revealed that *Monascus* spp. produced citrinin CIT, which was toxic to animal kidney, heart, and liver, and could cause deformities, carcinogenesis, and even induce gene mutations [9,10]. It has been reported that when the content of CIT is more than 50 μmol/L, it can cause the death of pig PK15 cells [11]. As a more widely contaminated fungal toxin, CIT can also synergize with other fungal toxins, such as ochratoxin A (OTA) and patulin (PTL), enhancing their toxicity. CIT restricts the application of *Monascus* spp. [12] and has a great negative effect on the production and export-oriented trade of *Monascus* spp. There is an urgent need to develop effective measures to prevent CIT contamination and minimize CIT synthesis in *Monascus* spp., as it has serious effects on several features, such as morphology and pigment synthesis [13], and can cause liver injury and reduced viability [14]. Studies have been devoted to elucidating the CIT biosynthetic pathway and its molecular regulatory mechanisms [15]. However, the CIT biosynthetic gene cluster is not fully defined, and its specific synthesis mechanism is not clear.

The *VeA* gene was identified as a global regulator in *Aspergillus nidulans* and is considered a direct homolog in fungal development and metabolism [16,17,18]. *VeA* has multiple functions due to changes in cell localization and the formation of different protein complexes [19,20]. Studies have proved that the VeA protein only exists in the fungal kingdom, which is a major member of the velvet family proteins [21]. The regulatory mechanism of the *VeA* protein in *Aspergillus nidulans*, *Neurospora crassa*, *Fusarium oxysporum*, *Aspergillus flavus*, and *Aspergillus fumigatus* has been widely studied, and the functions of the *VeA* gene in biological processes in fungi have also been well investigated, while the function of the *VeA* gene in *Monascus* spp. has rarely been reported [22,23,24]. We also hypothesize that the VeA protein in *Monascus* spp. plays a critical role in its growth and development, secondary metabolism, and virulence factors. Therefore, in this study, deletion of the *VeA* gene in *Monascus* M7 (*ΔVeA*) was constructed, and we also performed RNA sequencing analyses of the *ΔVeA* and WT strain to investigate the role of *VeA* in growth and development and CIT synthesis. It provides new ideas to effectively decipher the molecular mechanism of action of the VeA protein.

## 2. Materials and Methods

### 2.1. Strains, Plasmids, and Media

*M. ruber* M7 (CCAM 070120, Culture Collection of State Key Laboratory of Agricultural Microbiology, attached to the China Center for Type Culture Collection, Wuhan, China) was used as the parent to generate mutants. Plasmids pSKH and pCAMBIA3300 were kept in our laboratory for constructing recombinant vectors.

Potato dextrose agar (PDA), malt extract agar (MA), Czapek yeast extract agar (CYA), and 25% glycerol nitrate agar (G25N) were utilized for morphological characteristics. Potato Dextrose Broth (PDB) medium was used for the analysis of MPs and CIT production. The coculture-inducing medium was applied to generate mutants of *M. ruber* M7 [25]. Toselectmutants, PDA supplied with 30 µg/mL Hygromycin B (Sigma-Aldrich, Shanghai, China) was used to isolate neomycin-resistant transformants. All strains were maintained on PDA slants at 28 °C.

### 2.2. Cloning of the Wild-Type VeA Gene

The whole genome sequence of *M. ruber* M7 was provided by Chen’s team from Huazhong Agricultural University. According to the protein sequence of *Aspergillus nidulans* (AAD42946.1), the *VeA* gene in the *M. ruber* M7 genome was obtained by HMMER software (v3.4; http://hmmer.org/). The PCR amplification primers were designed (Table A1), and the entire *VeA* gene sequence was amplified from the genomic DNA of *M. ruber* M7. The target fragment was purified by the gel DNA small recovery kit (Magen, Guangzhou, China) and sent to Shanghai Sangon Biotech for sequencing. The sequencing result of the *VeA* gene was compared to the sequence published on NCBI by BLAST (v2.15.0+; https://blast.ncbi.nlm.nih.gov/Blast.cgi, accessed on 2 November 2025). The amino acid number, isoelectric point, and molecular weight of VeA protein were analyzed on the ExPASy website using the ProtParam tool (https://web.expasy.org/protparam/, accessed on 2 November 2025).

### 2.3. Construction of a VeA Gene Knockout Cassette

**Figure 1:**
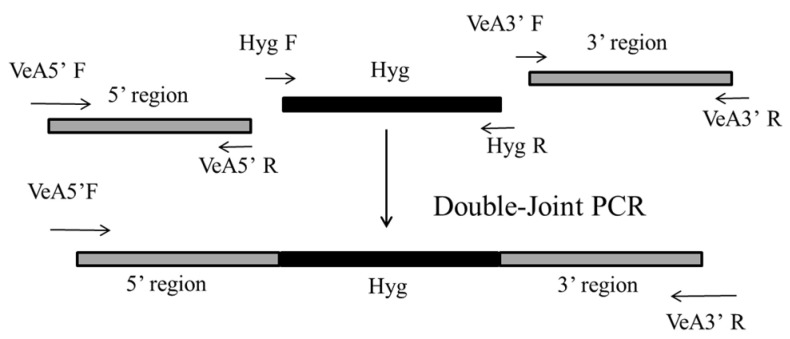
Construction of a *VeA* gene knockout cassette strategy by double-joint PCR. The two gray line segments are the 5′ flanking regions and 3′ flanking regions, homologous arms on the left and right sides of the *VeA* gene, and the gray line segments are the *Hyg* resistance genes. The primers amplified the 5′ flanking regions, the 3′ flanking regions, and *Hyg F*/*Hyg* R for *VeA* 5′ F/*VeA* 5′ R, *VeA* 3′ F/*VeA* 3′ R, and *Hyg F*/*Hyg* R, respectively.

The plasmid pSKH genome was used as a template to amplify the *Hyg* gene using primers *Hyg F*/*Hyg* R. The *VeA* gene 5′ and 3′ flanking regions were amplified from the genomic DNA of *M. ruber* M7. Using these amplified DNA sequences, the *VeA* gene deletion cassette was constructed by double-joint PCR [26]. The construction strategy is shown in Figure 1. The PCR system was 12.5 μL 2× Ftaq Master Mix, 5′ flanking regions DNA fragment, *Hyg* DNA fragment, and 3′ flanking regions DNA fragment in a molar ratio (1:2:1) to make a fusion system, hydrated to 25 μL. Double-Joint PCR program: 94 °C for 5 min, (94 °C for 15 s, 55 °C for 15 s, 72 °C for 8 min) × 15 cycles, 72 °C for 5 min, to obtain the knockout cassette. The fused PCR product (knockout cassette) was used as the template, and the primers *VeA* 5′ F/*VeA* 3′ R at both ends of the knockout cassette were used for amplification and enrichment of the knockout cassette. 0.8% agarose gel was used to separate the PCR products, and the knockout cassette was recovered by cutting the gel.

### 2.4. Construction of ΔVeA Knockout Vector

**Figure 2:**
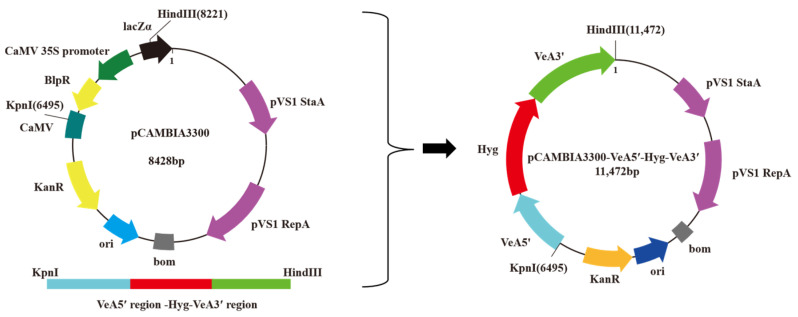
*VeA* gene knockout vector construction diagram.

Double digestion of the knockout cassette and plasmid vector pCAMBIA3300 using restriction endonucleases Hind III and Kpn I, respectively, and the knockout cassette was ligated between the polyclonal sites of plasmid vector pCAMBIA3300 under the action of T4 DNA ligase (Figure 2).

The *VeA* knockout cassette and the binary vector pCAMBIA3300 were separately double-digested with the restriction endonucleases Hind III and Kpn I. The digested products were separated by 0.8% agarose gel electrophoresis and then purified using a gel DNA small recovery kit (Magen, Guangzhou, China). The purified digested products were mixed with T4 DNA ligase and incubated at 16 °C for 4 h. A 5 μL aliquot of the ligation product was used to transform competent *Escherichia coli* DH5a cells. Randomly selected transformant colonies were verified by colony PCR with Hyg F/Hyg R as the specific primers. Plasmids were extracted from positive colonies using the plasmid DNA micro extraction kit (Magen, Guangzhou, China), followed by double digestion with Hind III and Kpn I. The plasmid with the correct enzyme digestion is the *VeA* knockout vector.

### 2.5. Agrobacterium-Mediated Transformation of Monascus ruber M7

The pCAMBIA3300-*VeA* knockout vector was transferred into *Agrobacterium tumefaciens* EHA105 receptor cells by the freeze-thaw method, and then the *ΔVeA* strain was achieved with the help of *Agrobacterium*-mediated T-DNA transformation *of Monascus rube* [27]. After initial screening of transformants using *Hyg* as a resistance screening marker, the genomes of *M. ruber*-positive transformants were extracted. Primers *VeA* CDS yz F/*VeA* CDS yz R, *VeA* 5′ F/*VeA* CDS yz R, *VeA* CDS yz F/*VeA* 3′ R, *VeA* 5′ F/*VeA* 3′ R, *Hyg* F/*Hyg* R, *VeA* 5′ F/*Hyg* R, and *Hyg* F/*VeA* 3′ R were used for PCR verification to screen *ΔVeA* strains. All primers used in this study are listed in Table A1.

### 2.6. Real-Time Quantitative PCR

The expression level of the *VeA* gene was measured by real-time fluorescence quantitative PCR (qPCR). Total RNA was extracted from WT and *ΔVeA* cultured on PDA medium at 28 °C for 3 days using TransZol Up Plus RNA Kit (TransGen Biotech, Beijing, China), Reverse Transcription of RNA into cDNA using the Evo M-MLV Reverse Transcription Premixed Tracer Kit (Accurate Blology, Hunan, China). Executed using PerfectStart Green qPCR SuperMix (TransGen Biotech, Beijing, China) for qPCR. The reference gene was glyceraldehyde-3-phosphate dehydrogenase (GAPDH). The qPCR program was: (95 °C 30 s × 1 cycles; (95 °C 5 s, 60 °C 30 s) × 40 cycles) to the Dissociation stage. The expression of related genes was calculated by the 2^−ΔΔCt^ method.

### 2.7. Differences in Growth and Development Between WT and ΔVeA

The spore suspensions of the WT and *ΔVeA* strains were inoculated onto the center of PDA, CYA, G25N, and MA media by aspirating 2 μL (1 × 10^5^ spores/mL), and incubated at 28 °C under dark conditions. Three replicates were set for each strain. After 7 days, the CX40 microscope (SUNNY, Ningbo, China) was used to observe the conidia and cleistothecia. After 10 days, Colony morphology was observed. Colony diameters of WT and *ΔVeA* strains cultured on PDA solid medium were determined by cross measurement at 3, 5, 7, 9, and 11 days of incubation.

### 2.8. The Biomass, Production of MPs, and CIT of WT and ΔVeA

The spore suspensions of 100 μL (1 × 10^5^ spores/mL) WT and *ΔVeA* strains were inoculated into 30 mL of PDB liquid medium, respectively. Three replicates were set for each strain and incubated at 28 °C for 5, 7, 9, 11, 13, and 15 days. The fermentation broth and mycelium were sampled and separated by filter paper filtration, and the mycelium was lyophilized to measure biomass and intracellular pigments. The filtrate was used for measuring extracellular pigments and CIT. The yields of yellow, orange, and red pigments were measured utilizing a UV spectrophotometer (HITA CHI U-3900, Tokyo, Japan) at wavelengths of 380, 470, and 520 nm, respectively [28]. The filtered fermentation broth was extracted with an equivalent volume of toluene-ethyl acetate-formic acid (7:3:1), centrifuged at 13,680× *g* for 10 min. The upper clarified organic phase was transferred to a clean beaker, evaporated in a water bath at 65 °C (or dried naturally), extracted with 80% methanol, and filtered through a 0.22 μm membrane. Then it was detected by an ultra-performance liquid chromatography fluorescence detector (UPLC-FLD). The detection conditions were as follows: Phenomenex C18 column (Phenomenex Luna, 2.1 mm × 100 mm, 1.7 μm), mobile phase: A, 0.1% formic acid in water; B, acetonitrile; elution program: 0–3 min, 90% A and 10% B; 3–10 min, 30% A and 70% B; 10.01–12 min, 10% A and 90% B; 12.01–15 min, 90% A and 10% B; column temperature was 40 °C; detection conditions: excitation wavelength, 330 nm; emission wavelength, 500 nm; flow rate: 0.3 mL/min; injection volume, 2 μL. The peak areas of CIT liquid phase were obtained, compared with the peak areas of CIT standards, and the contents of CIT in the fermentation were calculated.

### 2.9. RNA Extraction, Library Construction and RNA Sequencing

100 μL WT and *ΔVeA* spore suspension (10^5^ spores/mL) was inoculated on a PDA plate and cultured at 28 °C. Samples were taken on day 5 and day 11 of culture and frozen in liquid nitrogen at −80 °C for 1 h for total RNA extraction. Total RNA was extracted from WT and *ΔVeA* using TransZol Up Plus RNA Kit (TransGen Biotech, Beijing, China). All library construction and sequencing reactions were done at Sangon Biotech Co. RNA extraction, library construction, sequencing, data filtering, and quality control were performed according to the company’s operating guidelines. The reference genome is the genome of *M. ruber* M7 sequenced by Chen’s team from Huazhong Agricultural University. We also performed Gene Ontology (GO, https://geneontology.org/) and Kyoto Encyclopedia of Genes and Genomes (KEGG, https://www.kegg.jp/, accessed on 2 November 2025) pathway functional enrichment analyses.

### 2.10. Statistical Analysis

Experiments were repeated in triplicate, and all data were presented as the mean ± standard error. One-way analysis of variance in SPSS 24.0 was used to analyze whether the difference was significant using Duncan’s multiple-range test. The difference was considered significant at *p* < 0.05.

## 3. Results

### 3.1. The Analysis of the VeA Gene Sequence

The blast results showed 62.5% similarity between the *VeA* gene of this study and the *VeA* gene of *Monascus purpureus* (TQB74996.1) published in NCBI. The gene encodes 563 amino acids, with highly conserved regions. The predicted molecular weight of the protein is 62,634.53 Da, the total number of negatively charged residues was 55, the total number of positively charged residues was 71, the isoelectric point was 9.35, and the instability coefficient was 73.45.

### 3.2. Construction of ΔVeA Strain

#### 3.2.1. Construction of the *ΔVeA* Knockout Cassette

**Figure 3:**
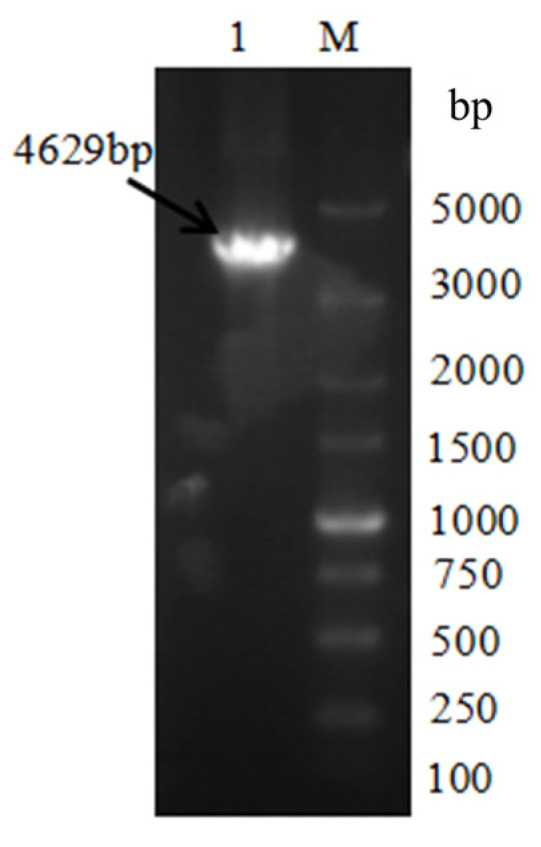
The *VeA* knockout cassette electrophoresis diagram. M is Marker, and lane 1 is the knockout cassette of 5′ region-*Hyg-VeA* 3′ region (4629 bp).

The fused PCR product (knockout cassette) was used as the template, and the knockout cassette was amplified and enriched with primer at both ends of the knockout cassette. The 0.8% agarose gel was used to separate the PCR product, and the cut gel was used to recover the knockout cassette (Figure 3).

#### 3.2.2. Integrating the Knockout Cassette into the Vector

**Figure 4:**
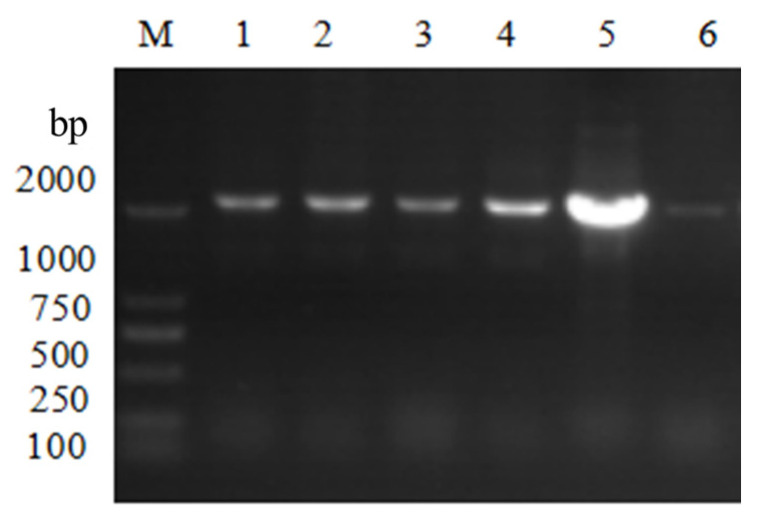
pCAMBIA3300-*VeA* knockout vector colony PCR verified electrophoretic diagram. M is Marker. Lanes 1, 2, 3, 4, and 5 are pC3300-*VeA* knockout carrier positive converters, lane 6 is non-positive converters.

**Figure 5:**
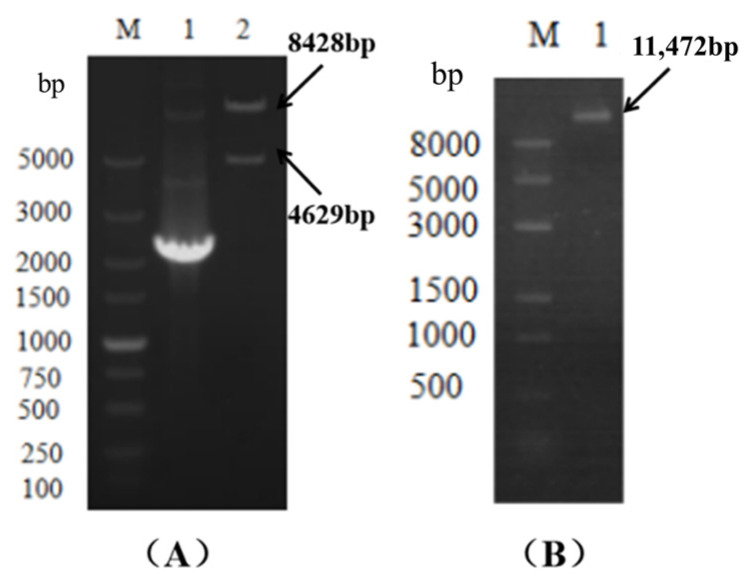
The pC3300-*VeA* knockout vector was verified by single- and double-enzyme digestion electrophoresis. M: Marker, lane 2 in (**A**) showed the electrophoresis diagram of pC3300-*VeA* knockout carrier double-enzyme digestion verification. (**B**) shows the electrophoresis of pC3300-*VeA* knockout vector with single-enzyme digestion verification.

Verified by colony PCR using *Hyg F*/*Hyg* R primers, and verified successfully that lane 5 was the positive transformant of *Escherichia coli* DH5a (Figure 4); extracted lane 5 positive transformant plasmid DNA was verified by single and double digestion using Hind III and Kpn I (Figure 5). The digestion verification results were correct, and the pCAMBIA3300-*VeA* knockout vector was successfully constructed.

#### 3.2.3. *Agrobacterium*-Mediated Transformation of *Monascus ruber* M7

**Figure 6:**
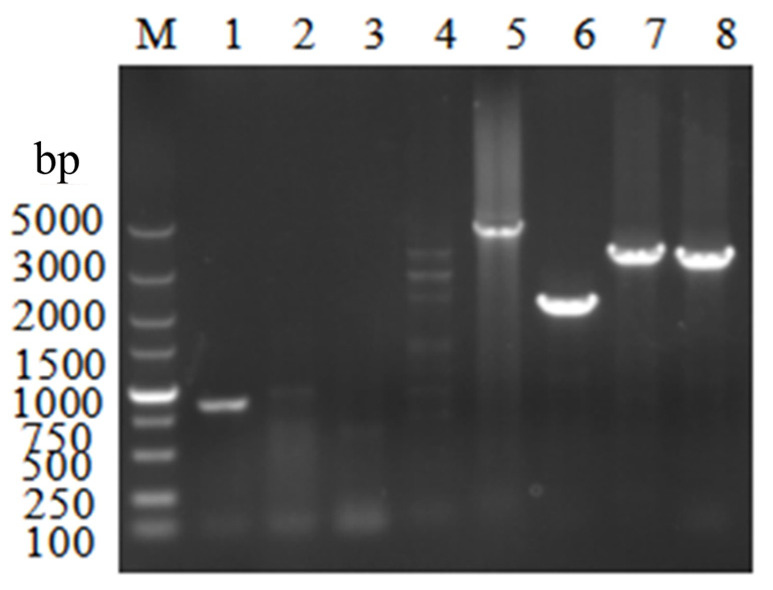
Validation of the *ΔVeA* strain by PCR. M: Marker. Lane 1 is the blank control (871 bp) amplified with *VeA* CDS yz *F*/*VeA* CDS yz R primers on the WT strain genome as template. Lanes 2–8 are the validation PCR amplified with the transforming sub-genome as template. Lanes 2–4 (cannot be expanded) are *VeA* CDS yz *F*/*VeA* CDS yz R (871 bp), *VeA* 5′ *F*/*VeA* CDS yz R (2107 bp), and *VeA* CDS yz *F*/*VeA* 3′ R (2068 bp). Lanes 5–8 are *VeA* 5′ *F*/*VeA* 3′ R (4629 bp), *Hyg F*/*Hyg* R (2137 bp), *VeA* 5′ F/Hyg R (3365 bp), and *Hyg F*/*VeA* 3′ R (3327 bp).

The positive transformants were isolated and purified to obtain purebred knockout transformants; their genomic DNA was extracted, and the selected knockout mutant strains were verified by PCR using seven primer pairs. The results are shown in Figure 6 (one of the positive transformants), and the *VeA* knockout strain was successfully constructed.

### 3.3. Expression of VeA Gene in WT and ΔVeA

**Figure 7:**
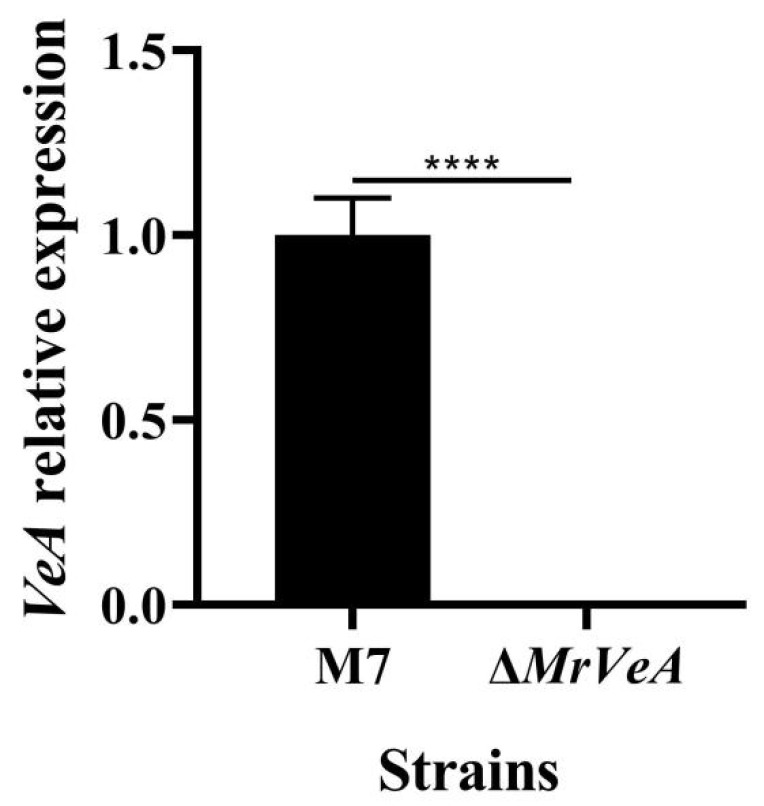
The relative expression of the *VeA* gene in the WT and the *ΔVeA* strain. The data are represented as the mean ± standard error (*n* = 3). **** *p* < 0.001.

The relative expression of the *VeA* gene in the WT and the *ΔVeA* strain on day 3 was detected by qPCR, and the results are shown in Figure 7. The *VeA* gene was barely expressed in the *ΔVeA* mutant strain, which was much lower than the expression of the *VeA* gene in the WT strain. It was confirmed that the *VeA* knockout vector was transcribed normally in *M. ruber* M7, and the knockout target was achieved.

### 3.4. Differences in Growth and Development Between WT and ΔVeA Strains

**Figure 8:**
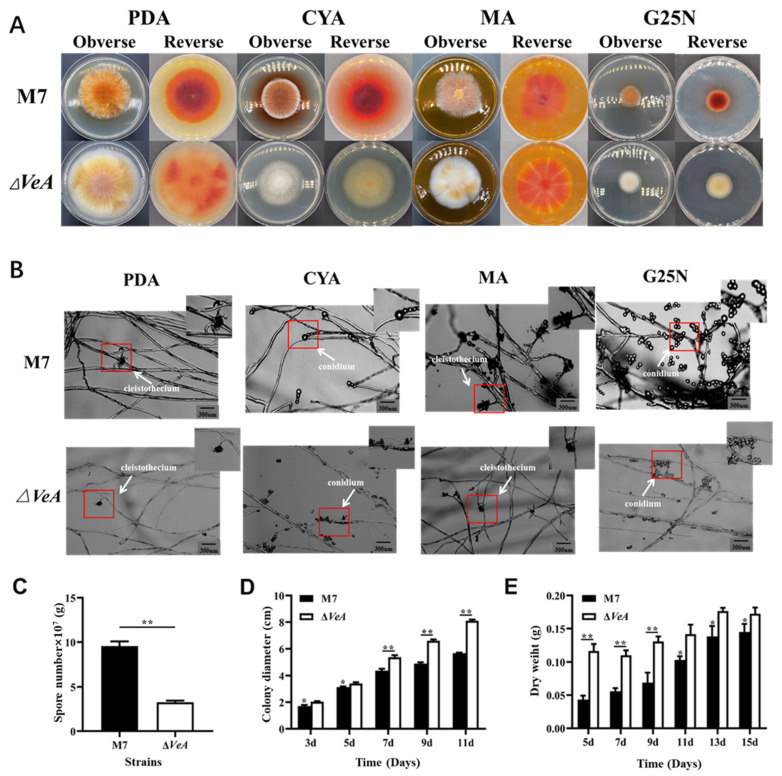
Phenotypic analysis of the WT and *ΔVeA* mutant strain. (**A**) colony morphologies on PDA, CYA, G25N, and MA media. (**B**) conidia and cleistothecia morphologies. The red box represents the enlarged portion. (**C**) numbers of conidia, (**D**) growth rate, and (**E**) biomass. The data are represented as the mean ± standard error (*n* = 3). * *p* < 0.05, ** *p* < 0.01.

The colony morphology of the WT and *ΔVeA* strain is shown in Figure 8A. The WT strain has a neat colony edge, with a large number of yellow-brown spores produced in the middle, and the mycelium at the edge of the colony was creamy white. In contrast, the *ΔVeA* strain had well-developed mycelium, less spore production, and neat colony edges. The mycelium morphology observed under the light microscope is shown in Figure 8B. The *ΔVeA* strain had dense mycelium with a longer length and a reduced closed capsule shell compared with the WT strain. The WT strain produced three times more conidia than the *ΔVeA* strain (Figure 8C). The growth rate of the *ΔVeA* strain was found to be significantly faster than that of the WT strain by measuring the colony diameter of the strain (Figure 8D). The biomass of the WT strain increased gradually with the extension of incubation time, while the biomass of the *ΔVeA* strain first increased rapidly and then stabilized, but was always heavier than the WT strain, with biomass increasing by 28.26% and 19.31% at 13 d and 15 d, respectively (Figure 8E).

### 3.5. The Capacity of Producing Pigment in WT and ΔVeA

**Figure 9:**
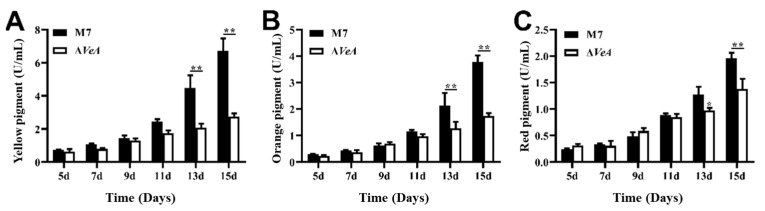
Analysis of the extracellular pigment production capacity of the WT and *ΔVeA* strain. (**A**) extracellular yellow pigment: primarily Monascin and Ankaflavin, polyketide-derived secondary metabolites. (**B**) extracellular orange pigment: typically Monascorubramine, often an intermediate between yellow and red pigments. (**C**) extracellular red pigment: mainly Monascorubrin and Rubropunctatin, which are azaphilone-type polyketides. The data are represented as the mean ± standard error (*n* = 3). * *p* < 0.05, ** *p* < 0.01.

**Figure 10:**
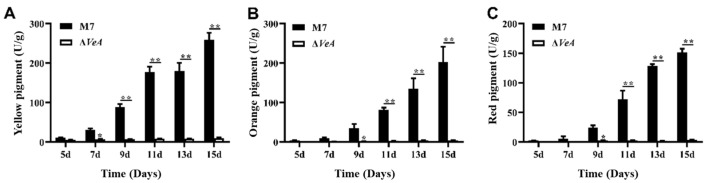
Analysis of the intracellular pigment production capacity of the WT and *ΔVeA* strain. (**A**) intracellular yellow pigment: Primarily Monascin and Ankaflavin, polyketide-derived secondary metabolites. (**B**) intracellular orange pigment: typically Monascorubramine, often an intermediate between yellow and red pigments. (**C**) intracellular red pigment: mainly Monascorubrin and Rubropunctatin, which are azaphilone-type polyketides. The data are represented as the mean ± standard error (*n* = 3). * *p* < 0.05, ** *p* < 0.01.

The *M. ruber* M7 followed the pattern of the highest yield of yellow pigment, followed by orange pigment, and the lowest yield of red pigment, as shown in Figure 9 and Figure 10. The extracellular pigments of the WT and the *ΔVeA* strain increased continuously from 5 to 15 d, but the production of extracellular yellow pigment of the *ΔVeA* strain decreased by 54.7% and 64.4% at 13 d and 15 d, respectively, compared to the WT strain, as shown in Figure 9A. The production of extracellular orange pigment of the *ΔVeA* strain decreased by 39.7% and 55.3% at 13 d and 15 d, respectively, compared to the WT strain, as shown in Figure 9B. The production of extracellular red pigment of the *ΔVeA* strain decreased by 39.7% and 55.3% at 13 d and 15 d, respectively, compared to the WT strain. The production of extracellular red pigment of the *ΔVeA* strain decreased by 17.5% and 27.7% at 13 d and 15 d, respectively, compared to the WT strain, as shown in Figure 9C.

The intracellular MPs were measured using UV-Vis after 30 min ultrasonic extraction of the MPs with 80% methanol (Figure 10). Intracellular pigment of the WT strain increased continuously from 5 d to 15 d. The intracellular pigment of the *ΔVeA* strain did not change significantly, and the pigment yield was greatly reduced compared to the WT strain.

### 3.6. The CIT Production of WT and ΔVeA

**Figure 11:**
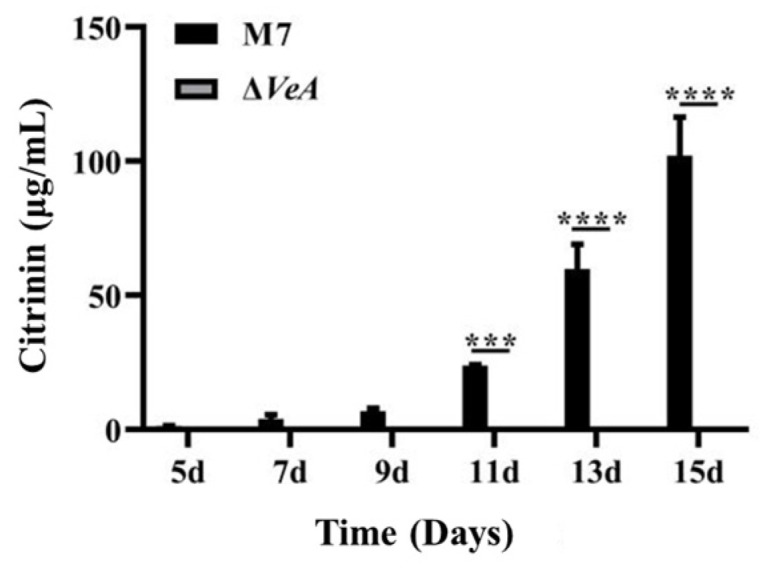
Analysis of the CIT production capacity of the WT strain and the *ΔVeA* strain. The data are represented as the mean ± standard error (*n* = 3)., *** *p* < 0.005, ****p < 0.001.

The CIT production ability of WT strain and *ΔVeA* strains was analyzed by HPLC at 28 °C in a static culture protected from light, and the results are shown in Figure 11. The CIT production ability of the WT strain increased with the increase of culture time, and after 15 d of culture, the WT strain produced CIT up to 40.6 ± 6.4 μg/mL, and all three *ΔVeA* mutant strains did not produce CIT.

### 3.7. RNA Sequencing Data Analysis

A total of 207,210,840 reads were obtained from the transcriptome sequencing of wild-type, 5-day and 11-day deletion strains. 201,834,468 clean reads were obtained after quality control, and the error rate of each sample was less than 0.05%, and the sequence quality control parameters Q20 and Q30 were above 95%, indicating that the sequencing results were good enough for the next step analysis.

### 3.8. DEGs Expression Analysis

#### 3.8.1. DEGs Statistics

**Figure 12:**
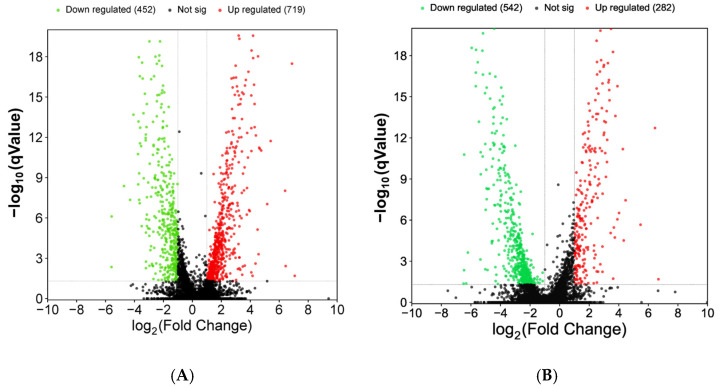
Volcano plots of DEGs in WT vs. *ΔVeA*. (**A**) DEGs at 5 days of cultivation; (**B**) DEGs at 11 days of cultivation. The horizontal axis is the fold-change value of gene expression difference between different groups of samples, and the vertical axis is the value representing the gene expression. In the graph, each dot represents a gene, where red indicates up-regulated genes, green indicates down-regulated genes, and black indicates non-differential genes.

The DEGs expression is shown in Figure 12. The knockout was significantly up-regulated in 719 genes and down-regulated in 452 genes compared to the wild type at 5 days. The knockout had 284 genes up-regulated and 542 genes down-regulated compared to the wild type at 11 days. (Figure 12).

#### 3.8.2. Enrichment Analysis of Candidate Genes

**Figure 13:**
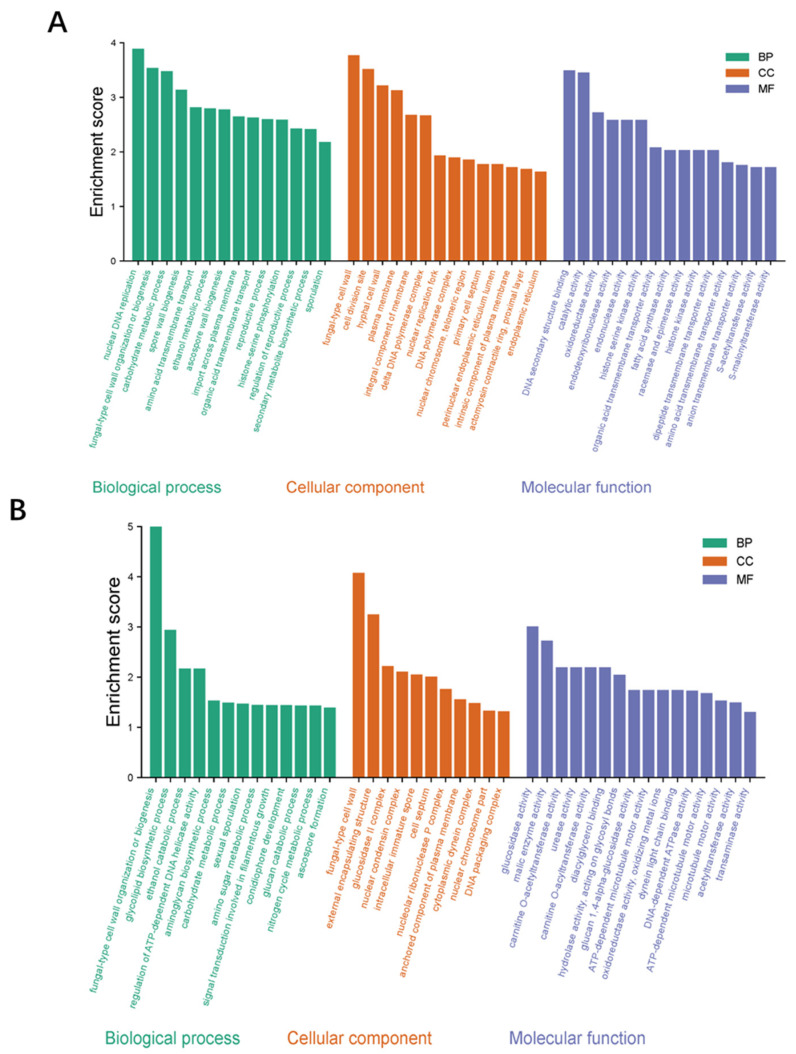
Gene function analysis of DEGs in WT vs. *ΔVeA*. The Gene function analysis by DEGs on the 5th d (**A**) and 11th d (**B**). The plot is divided into three categories: Biological Process (BP, green), Cellular Component (CC, orange), and Molecular Function (MF, purple).

**Figure 14:**
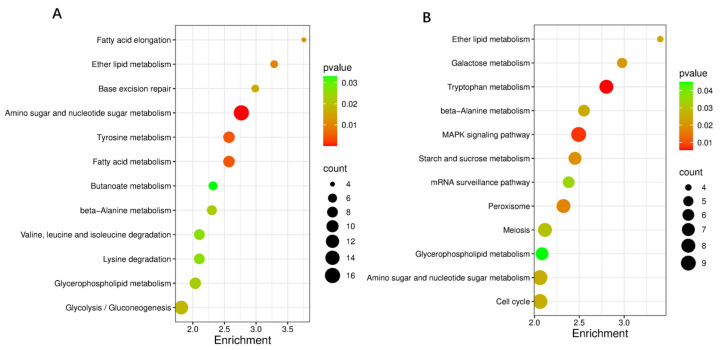
KEGG enrichment analysis of DEGs in WT vs. *ΔVeA*. The KEGG pathways enriched by DEGs on the 5th d (**A**) and 11th d (**B**). The size of the dot indicates the number of DEGs, and a smaller *p*-value indicates a more significant enrichment.

The GO analysis of the obtained DEGs mainly included 3 categories: biological process, cellular component, and molecular function (Figure 13). Among the biological processes, the ethanol metabolic process, fungal-type cell wall organization, and sporulation were mainly involved. Among the cellular components, the main functions were focused on the spore wall, the hyphal cell wall, and the cell septum. Among the molecular functions, the main functions were focused on metabolic process, catalytic activity, and transport activity. In terms of KEGG pathway enrichment, at the early cultivation stage (Figure 14A), DEGs are mainly enriched in tyrosine metabolism, Glycolysis/Gluconeogenesis, and fatty acid metabolism. During the late culture stage (Figure 14B), DEGs were mainly enriched in the tryptophan metabolism pathway, starch and sucrose metabolism, and the MAPK signaling pathway.

## 4. Discussion

The *VeA* gene is a global regulator of developmental and secondary metabolites in filamentous fungi. We constructed a mutant strain, *ΔVeA*, using the Agrobacterium-mediated transformation technique to investigate the *VeA* gene on growth and development, MPs, and CIT synthesis in *M. ruber* M7. Our results indicate that the *VeA* gene is involved in the regulation of developmental processes, mycelial growth, and pigment production in *M. ruber* M7.

*M. ruber* is a small saprophytic filamentous fungus, isolated and named by the Western scholar vanTieghem from oriental fermented foods in 1884. A series of studies have described and documented 36 species [29]. Sato was the first to classify *M. ruber* using colony characteristics on different media based on microscopic morphology and initially established a search list for *Aspergillus erythropolis* [30]. *VeA* belongs to the filamentous regulatory system, which regulates the development and secondary metabolism of many fungi. Previous studies found that the lack of *VeA* homologs in *Aspergillus fumigatus* and *Aspergillus parasiticus* resulted in reduced conidial production. Wang found a nearly twofold reduction in the length and width of conidia and vesicles compared to the wild strain by knocking out the *VeA* gene of *Aspergillus niger* [31]. A large number of studies have shown that the *VeA* gene affects the growth and development of filamentous fungi [19]. In our present study, the deletion of the *VeA* gene increased the growth rate and biomass of *M. ruber* M7, but its sexual and asexual reproduction were inhibited.

MPs are one of the important secondary metabolites produced by *M. ruber* during the fermentation process and are a mixture of a class of polyketides. *M. ruber* produces at least six alcohol-soluble pigments [32], which are divided into three groups of pigments, including yellow pigment, orange pigment, and red pigment [33]. The extracellular pigment of the WT and *ΔVeA* strains increased continuously from 5 to 15 days, but the extracellular pigment of the *ΔVeA* strain was always lower than that of the WT strain, and the maximum difference was reached at day 15. The results suggested that the *VeA* gene is able to regulate the production of extracellular pigments in *M. ruber* M7. The amount of yellow and orange pigments produced by the *ΔVeA* strain significantly decreased at 15 days compared with the WT strain, indicating that the *VeA* gene has a more significant regulatory effect on the production of extracellular yellow and orange pigments in *M. ruber*, and has relatively less effect on the production of extracellular red pigments. Similar results were also reported by Cary et al. [34]. The *ΔVeA* intracellular pigment, compared to WT, was consistently maintained at low levels and produced almost no intracellular pigment. The above study showed that MPs increased with increasing culture time, but the *ΔVeA* had almost no ability to produce MPs.

The *VeA* gene is a key gene for CIT synthesis in *M. ruber* M7. CIT in WT strain increased with time and reached a maximum at 15 days. The deletion of *VeA* gene completely stopped the expression of CIT, indicating that the *VeA* gene can positively regulate CIT biosynthesis in *M. ruber* M7. In most cases, the *VeA* is a positive regulator of secondary metabolite biosynthesis, such as aflatoxin in *Aspergillus parasiticus* and *Aspergillus flavus* [35], cypyridinic acid and aflatoxin in *Aspergillus flavus* [23], and ochratoxin in *Aspergillus carbonarius* [36]. These studies suggest that the *VeA* gene is an important regulator of toxin synthesis in *Aspergillus*.

Transcriptome sequencing data were consistent with the phenotypic changes observed in *ΔVeA*. A number of significantly up- and down-regulated genes were detected by transcriptome analysis. This shows that *VeA* regulates the expression of a large number of genes in *Monascus*, which also makes *VeA* occupy an important position in the whole gene expression regulation network. GO analysis showed that DEGs were mainly concentrated in cell process, metabolic process, catalytic activity, and transport activity. The DEGs in the *Aspergillus nidulans ΔVeA* mutant were significantly enriched in primary metabolic pathways, such as translation and amino acid metabolism, and were also involved in secondary metabolic processes related to catalytic activity and transport activity [18]. This characteristic was highly consistent with the GO classification results of the *Monascus VeA* gene knockout strain. In terms of KEGG pathway enrichment, at the early cultivation stage, DEGs are mainly enriched in tyrosine metabolism and fatty acid metabolism; these two pathways serve as core supports for cell growth, including protein synthesis and membrane structure construction [37,38]. During the late culture stage, DEGs were mainly enriched in the tryptophan metabolism pathway and the MAPK signaling pathway. The former is the core precursor synthesis pathway of MPs [39], while the latter is involved in fungal sexual reproduction and stress response as a key signaling pathway [40]. Transcriptome data further indicated that *VeA* played an important regulatory role in the production of MPs and CIT in *M. ruber* M7. This is of great significance for understanding the complex regulatory mechanisms of MPs and mycotoxin citrinin.

## Data Availability

Data will be made available on request.

## Appendix A

**Table A1:** Primers were used in this study.

Primer	Sequence (5′–3′)	Size (bp)	Function
*VeA 5′ F*	CCCAGCTGTGGTTCTCTTTTCCGTCTCCAAC	1331	Cloning 5′ flanking regions
*VeA 5′ R*	CAATATCATCTTCTGTCGG AGCTCAGTCGATATCGACCCTC		
*Hyg F*	GTCGACAGAAGATGATATTG	2137	Cloning
*Hyg R*	CTAGAAAGAAGGATTACCTC		
*VeA 3′ F*	GAGGTAATCCTTCTTTCTAGCTCAGACACCCATAAACATGATGC	1217	Cloning 3′ flanking regions
*VeA 3′ R*	GGGGTACCAGCG CAAATCAACGGGTTCCATTAC		
*VeA CDS yz F*	CATCTGTACGAGGAGACCAAGG	871	Cloning
*VeA CDS yz R*	AACGAGAAGCGGGTGACGGGAG		
*VeA CDS F*	ATGGCGGCCAGAGCACCAATG	1692	Cloning
*VeA CDS R*	CTATGGATTTGGGTGGATTTTC		
*q* M7 *F*	CAAGCTCACTGGCATGTCTATG	301 bp	qPCR
*q* M7 *R*	AAGTTCGAGTTGAGGGCGATA		
*qVeA F*	GAGTCGGTCTTGTCTAAG	100 bp	qPCR
*qVeA R*	CACTAGGGTACGTTGTTC		
*GAPDH F*	CAAGCTCACTGGCATGTCTATG	301 bp	reference gene
*GAPDH R*	AAGTTCGAGTTGAGGGCGATA		

Note: The underlined sequence in Table A1 represents the restriction site, and the wave line sequence represents the *Hyg* primer sequence.

## References

[1] Ignatius S., Endang K., Elok Z., Susana R., Ira N., Andreas A., Nathania I., Bo Z.B. (2021). Utilization of agro-industrial by-products in *Monascus* fermentation: A review. Bioresour. Bioprocess..

[2] Ji-Eun L., Gi M.S., Wook H.S., Jae-Hwan K., Gwang-Ho K., Ye-Rang Y. (2023). Anti-obesity effects of kimchi with red yeast rice in 3T3-L1 adipocytes and high-fat diet-induced obese mice. J. Funct. Foods.

[3] Ye F., Zhang C., Liu S., Liu X., Liu J., Guo T., Lu D., Zhou X. (2024). Optimization of medium compositions and X-ray irradiation to enhance monacolin K production by *Monascus purpureus* in submerged fermentation. Process Biochem..

[4] Zhou J., Pan Q., Xue Y., Dong Y., Chen Y., Huang L., Zhang B., Liu Z.Q., Zheng Y. (2024). Synthetic biology for *Monascus*: From strain breeding to industrial production. Biotechnol. J..

[5] Feng Y., Yu X. (2020). Perspectives on Functional Red Mold Rice: Functional Ingredients, Production, and Application. Front. Microbiol..

[6] Wu M.H., Wang Q.Q., Zhang H., Pan Z.Y., Zeng Q.L., Fang W.Z., Mao J.L., Li J.P., Wu H., Qiu Z.P. (2023). Performance and mechanism of co-culture of *Monascus purpureus*, *Lacticaseibacillus casei*, and *Saccharomyces cerevisiae* to enhance lovastatin production and lipid-lowering effects. Bioprocess Biosyst. Eng..

[7] Fan X.C., Han J., Zhang F., Chen W.S. (2023). Red yeast rice: A functional food used to reduce hyperlipidemia. Food Rev. Int..

[8] Huang Z., Chen L., Xiao L., Ye Y., Mo W., Zheng Z., Li X. (2024). *Monascus*-fermented quinoa alleviates hyperlipidemia in mice by regulating the amino acid metabolism pathway. Food Funct..

[9] Ivana B., Katarina Z., Anna K., Marcela C. (2016). Toxicological properties of mycotoxin citrinin. J. Microbiol. Biotechnol. Food Sci..

[10] Blanc P.J., Laussac J.P., Bars J.L., Bars P.L., Loret M.O., Pareilleux A., Prome D., Prome J.C., Santerre A.L., Goma G. (1995). Characterization of monascidin A from *Monascus* as citrinin. Int. J. Food Microbiol..

[11] Rumora L., Domijan A., Grubišić T., Klarić M. (2014). Differential activation of MAPKs by individual and combined ochratoxin A and citrinin treatments in porcine kidney PK15 cells. Toxicon.

[12] López-Berges M.S., Concepción H., Michael S., Katja S., Javier C., Josep G., Di Pietro A. (2013). The velvet complex governs mycotoxin production and virulence of *Fusarium oxysporum* on plant and mammalian hosts. Mol. Microbiol..

[13] Zhang X., Chen W., Wang C. (2025). Regulation of citrinin biosynthesis in *Monascus purpureus*: Impacts on growth, morphology, and pigments production. Food Microbiol..

[14] Wu J., Yang C., Yang M., Liang Z., Wu Y., Kong X., Fan H., Wang S., Ning C., Xiao W. (2022). The role of ER stress and ATP/AMPK in oxidative stress meditated hepatotoxicity induced by citrinin. Ecotoxicol. Environ. Saf..

[15] He Y., Zhu L., Dong X., Li A., Xu S., Wang L., Shao Y. (2023). Metabolic Regulation of Two *pksCT* Gene Transcripts in *Monascus ruber* Impacts Citrinin Biosynthesis. J. Fungi.

[16] Calvo A.M. (2008). The *VeA* regulatory system and its role in morphological and chemical development in fungi. Fungal Genet. Biol..

[17] Hyoun-Young K., Kap-Hoon H., Mimi L., Miae O., Hee-Seo K., Xie Z., Dong-Min H., Kwang-Yeop J., Hwa K.J., Keon-Sang C. (2009). The *VeA* gene is necessary for the negative regulation of the *VeA* expression in *Aspergillus nidulans*. Curr. Genet..

[18] Moon H., Lee M.-K., Bok I., Bok J.W., Keller N.P., Yu J.-H. (2023). Unraveling the Gene Regulatory Networks of the Global Regulators *VeA* and *LaeA* in *Aspergillus nidulans*. Microbiol. Spectr..

[19] Zhang J., Chen H., Sumarah M.W., Gao Q., Wang D., Zhang Y. (2018). The *VeA* gene acts as a positive regulator of conidia production, ochratoxin A biosynthesis, and oxidative stress tolerance in *Aspergillus niger*. J. Agric. Food Chem..

[20] El Hajj Assaf C., Snini S.P., Tadrist S., Bailly S., Naylies C., Oswald I.P., Lorber S., Puel O. (2018). Impact of *VeA* on the development, aggressiveness, dissemination and secondary metabolism of *Penicillium expansum*. Mol. Plant Pathol..

[21] Tan Y., Wang H., Wang Y., Ge Y., Ren X., Ren C., Wang Y., Ren X., Liu Y., Liu Z. (2018). The role of the *VeA* gene in adjusting developmental balance and environmental stress response in *Aspergillus cristatus*. Fungal Biol..

[22] Belén L., Luis G., AnaRosa B. (2022). Ochratoxin A Defective *Aspergillus carbonarius* Mutants as Potential Biocontrol Agents. Toxins.

[23] Xu J., Jiang M., Wang P., Kong Q. (2024). The Gene *vepN* Regulated by Global Regulatory Factor *VeA* That Affects Aflatoxin Production, Morphological Development and Pathogenicity in *Aspergillus flavus*. Toxins.

[24] Dhingra S., Lind A.L., Lin H.C., Tang Y., Rokas A., Calvo A.M. (2013). The fumagillin gene cluster, an example of hundreds of genes under *VeA* control in *Aspergillus fumigatus*. PLoS ONE.

[25] Zwiers L.H., De Waard M.A. (2001). Efficient *Agrobacterium tumefaciens*-mediated gene disruption in the phytopathogen *Mycosphaerella graminicola*. Curr. Genet..

[26] Shao Y., Ding Y., Zhao Y., Yang S., Xie B., Chen F. (2009). Characteristic analysis of transformants in T-DNA mutation library of *Monascus ruber*. World J. Microbiol. Biotechnol..

[27] Li L., He L., Lai Y., Shao Y., Chen F. (2014). Cloning and functional analysis of the *Gβ* gene Mgb1 and the *Gγ* gene Mgg1 in *Monascus ruber*. J. Microbiol..

[28] Meng C., Zhou Y., Gao Z., Liu J., Chen F. (2024). mrvam7, a conserved SNARE gene involved in vacuolar fusion, is required for development and secondary metabolism in *Monascus ruber* M7. Food Biosci..

[29] Hawksworth D., Pitt J. (1983). New taxonomy for *Monascus* species based on cultural and microscopical characters. Aust. J. Bot..

[30] Shao Y., Lei M., Mao Z., Zhou Y., Chen F. (2014). Insights into *Monascus* biology at the genetic level. Appl. Microbiol. Biotechnol..

[31] Wang F., Dijksterhuis J., Wyatt T., Wsten H.A.B., Bleichrodt R.J. (2015). *VeA* of *Aspergillus niger* increases spore dispersing capacity by impacting conidiophore architecture. Antonie Leeuwenhoek.

[32] Silveira S.T., Daroit D.J., Brandelli A. (2008). Pigment production by *Monascus purpureus* in grape waste using factorial design. LWT—Food Sci. Technol..

[33] Ghosh S., Dam B. (2020). Genome shuffling improves pigment and other bioactive compound production in *Monascus purpureus*. Appl. Microbiol. Biotechnol..

[34] Cary J.W., Harris-Coward P.Y., Ehrlich K.C., Di Mavungu J.D., Malysheva S.V., De Saeger S., Dowd P.F., Shantappa S., Martens S.L., Calvo A.M. (2014). Functional characterization of a *VeA* -dependent polyketide synthase gene in *Aspergillus flavus* necessary for the synthesis of asparasone, a sclerotium-specific pigment. Fungal Genet. Biol..

[35] Duran R.M., Cary J.W., Calvo A.M. (2007). Production of cyclopiazonic acid, aflatrem, and aflatoxin by *Aspergillus flavus* is regulated by *VeA*, a gene necessary for sclerotial formation. Appl. Microbiol. Biotechnol..

[36] Crespo-Sempere A., Marín S., Sanchis V., Ramos A.J. (2013). *VeA* and *LaeA* transcriptional factors regulate ochratoxin A biosynthesis in *Aspergillus carbonarius*. Int. J. Food Microbiol..

[37] Boyce K.J., McLauchlan A., Schreider L., Andrianopoulos A. (2015). Intracellular growth is dependent on tyrosine catabolism in the dimorphic fungal pathogen *Penicillium marneffei*. PLoS Pathog..

[38] Campanella J.E.M., Rosa L.T., Malavazi I. (2025). FungalΔ9-fatty acid desaturase: A unique enzyme at the core of lipid metabolism in *Aspergillus fumigatus* and a promising target for the search for antifungal strategies. mBio.

[39] Huang D., Wang Y., Zhang J., Xu H., Bai J., Zhang H., Jiang X., Yuan J., Lu G., Jiang L. (2021). Integrative Metabolomic and Transcriptomic Analyses Uncover Metabolic Alterations and Pigment Diversity in *Monascus* in Response to Different Nitrogen Sources. mSystems.

[40] Sánchez S.C., Villanueva S.M., Gutiérrez G., Cánovas D., Corrochano L.M. (2024). *VE-1* regulation of MAPK signaling controls sexual development in *Neurospora crassa*. mBio.

